# Assessment of Changes in the Oral Microbiome That Occur in Dogs with Periodontal Disease

**DOI:** 10.3390/vetsci8120291

**Published:** 2021-11-27

**Authors:** Rodrigo Santibáñez, Camila Rodríguez-Salas, Carla Flores-Yáñez, Daniel Garrido, Pamela Thomson

**Affiliations:** 1 Departamento de Ingeniería Química y Bioprocesos, Facultad de Ingeniería, Pontificia Universidad Católica, Santiago 8940000, Chile; rlsantibanez@uc.cl (R.S.); dgarridoc@ing.puc.cl (D.G.); 2 Laboratorio de Microbiología Clínica y Microbioma, Escuela de Medicina Veterinaria, Facultad de Ciencias de la Vida, Universidad Andrés Bello, Santiago 8370134, Chile; camnise@gmail.com; 3 Clínica Veterinaria Los Avellanos, Santiago 8380239, Chile; veterinarialosavellanos@gmail.com

**Keywords:** oral microbiome, periodontal disease, *Porphyromonas*, 16S rRNA

## Abstract

The oral microbiome in dogs is a complex community. Under some circumstances, it contributes to periodontal disease, a prevalent inflammatory disease characterized by a complex interaction between oral microbes and the immune system. *Porphyromonas* and *Tannerella* spp. are usually dominant in this disease. How the oral microbiome community is altered in periodontal disease, especially sub-dominant microbial populations is unclear. Moreover, how microbiome functions are altered in this disease has not been studied. In this study, we compared the composition and the predicted functions of the microbiome of the cavity of healthy dogs to those with from periodontal disease. The microbiome of both groups clustered separately, indicating important differences. Periodontal disease resulted in a significant increase in *Bacteroidetes* and reductions in *Actinobacteria* and *Proteobacteria*. *Porphyromonas* abundance increased 2.7 times in periodontal disease, accompanied by increases in *Bacteroides* and *Fusobacterium.* It was predicted that aerobic respiratory processes are decreased in periodontal disease. Enrichment in fermentative processes and anaerobic glycolysis were suggestive of an anaerobic environment, also characterized by higher lipopolysaccharide biosynthesis. This study contributes to a better understanding of how periodontal disease modifies the oral microbiome and makes a prediction of the metabolic pathways that contribute to the inflammatory process observed in periodontal disease.

## 1. Introduction

Periodontal disease is an inflammatory, multifactorial disease that affects the tissues that support dental pieces. It involves complex interactions between microorganisms in the oral cavity and the host immune response [1]. In dogs, it is one of the most prevalent diseases worldwide, affecting around 70% of canine patients [2,3,4,5]. The microbiome of the canine oral cavity is diverse and complex. It comprises the microbiota of different niches such as oral mucosa, tongue, saliva, supragingival and subgingival plaque [6]. Each of these niches is different in its composition, and together they create a unique ecosystem. When this microbiome suffers certain imbalances, a dysbiosis state generates a favorable environment for pathogenic microorganisms, increasing the virulence of microorganisms present [7,8]. These alterations exacerbate the immune response of the host, contributing to chronic inflammatory states and giving rise to the pathologies such as periodontal disease [9]. The etiology of this disease involves multiple factors, including the dental plaque and the community of microorganisms present at the dental surface. These bacteria establish a biofilm, colonizing the gingival grooves and the root surface of the tooth, sometimes resulting in the loss of dental pieces [10]. Tartar or dental calculus is generated when this biofilm is mineralized, and despite not being the leading cause of inflammation, it acts as a retention factor for microorganisms in the biofilm [11,12,13]. In humans, as in dogs and cats, periodontal disease and its pathogenesis have been strongly related to microorganisms such as *Porphyromonas* sp. [14,15,16,17]. Other research indicates that *Porphyromonas gulae*, *Tannerella forsythia,* and *Campylobacter rectus* are dominant periodontal pathogens in dogs [18]. Certain studies have addressed the differences in the composition of the microbiome of the canine oral cavity, both healthy and pathological, elucidating important information to understand the state of dysbiosis that periodontal disease entails. The presence of Gram-negative bacteria has been associated with healthy dogs, and an increase in Gram-positive bacteria has been associated with dogs with moderate periodontal disease [6]. On the contrary, it has been observed that the increase of anaerobic Gram-negative bacteria in the supragingival and subgingival plaque is related to the release of enzymes and endotoxins during the formation of periapical lesions [19].

How the oral microbiome is altered in dogs is unclear, and the impact of periodontal disease on sub-dominant microbial populations is unclear. The objective of this study was to compare the composition and the predicted functions of the microbiome of the cavity of healthy dogs to those suffering from periodontal disease using 16S rRNA sequencing.

## 2. Materials and Methods

### 2.1. Subjects and Inclusion Criteria

This study was approved by the Bioethics Committee at the Veterinary Clinic Los Avellanos (Approval Certificate HCVLA-010) and was carried out in one veterinary clinic, located in Independencia, Metropolitan Region, Chile (S33°24′54.4″ O70°39′56.7″). Samples were collected during the month of January 2021. Targeted sampling was performed to select 24 dogs, 12 with gingivitis or periodontitis and 12 without periodontal disease. Dogs older than two years were included, without distinction of breed or sex, without underlying pathologies and without antibiotic treatment at least three months before sampling.

The clinical examination of the oral cavity was performed by the same veterinarian, with prior authorization by informed consent. The presence or absence of gingivitis or periodontitis, gingival index (mild, moderate, severe) [20], dental calculus, and tooth exfoliation was evaluated during the clinical examination. In the group of healthy animals, all the assessed clinical signs were absent. In the periodontitis group, there were at least 4/6 clinical signs for inclusion. Data such as age, sex, race, body condition score (BS) [21] and type of diet were recorded for each patient (Table 1).

### 2.2. Analysis of the Oral Cavity Microbiome

The samples were obtained by swabbing the gingival margin of the right maxillary fourth premolar in the oral cavity, as previously described [22]. The swab was deposited in an Eppendorf tube with 1 mL of RNA later (Sigma Aldrich, St Louis, MO, USA). Before DNA extraction (Quick-DNA Fecal/Soil Microbe Miniprep Kit, Zymo Research, Irvine, CA, USA), each tube with its swab was placed in the disruption for 5 min using a Disruptor Genie device (Scientific Industries, Bohemia, NY, USA). DNA samples were diluted to 20 ng/µL in nuclease-free water (NanoDrop 2000c; Thermo Fisher Scientific, Waltham MA, USA). DNA samples were submitted for Illumina MiSeq sequencing to the DNA Sequencing Services at Molecular Research (MR-DNA, Shallowater, TX, USA). The variable region V3-V4 gene of the 16S rRNA was amplified using primers 341F and 785R. A barcode was added to the forward primer for pooling multiple samples. The reaction was run for 30 cycles using the HotStarTaq Plus Master Mix Kit (Qiagen, Hilden, Germany). DNA samples were pooled and purified using Ampure XP microspheres (Agencourt Bioscience Corporation, Boston, MA, USA). DNA libraries were prepared using the TruSeq DNA LT Sample Preparation Kit (Illumina, San Diego, CA, USA) following the manufacturer’s instructions. Sequencing was performed using the MiSeq platform (Illumina, San Diego, CA, USA).

### 2.3. Bioinformatics Analyses

Processed sequences were uploaded to the European Nucleotide Archive under the project code PRJEB47716. Bioinformatics analyses were done as previously described [23] with modifications. Sequences were filtered by quality and trimmed to 250 nucleotides and used to infer Amplicon Sequence Variants (ASVs) employing the DADA2 v1.10 R package [24]. Taxonomy was assigned utilizing the SILVA database version 132 [25,26] and a Naïve Bayesian classifier [27]. The abundance of metabolic pathways and enzymes were inferred from the ASV table using the PICRUSt2 python package [28] and the MetaCyc database [29]. Differences in the relative abundance of taxa were assessed with the non-parametric Mann-Whitney *U*-test [30], and differences in the abundance of metabolic functions and pathways were evaluated with the Linear Discriminant Analysis Effect Size (LEfSe) method [31]. The significance level for all statistical analyses was *p*-value < 0.05.

## 3. Results

The oral microbiome of 24 adult dogs was analyzed in this study. We included 12 dogs without periodontitis (S) and 12 animals with periodontal disease (P). The latter presented significant signs of gingivitis, erythema, gingival bleeding, tartars, or halitosis (Table 1; *p* < 0.05). Both groups were not different regarding their body scores (BS), but the dog’s group without gingivitis or periodontitis had a younger age than the other group (Table 1; *p* < 0.05). None of the included subjects had oral cavity hygiene habits.

**Figure 1:**
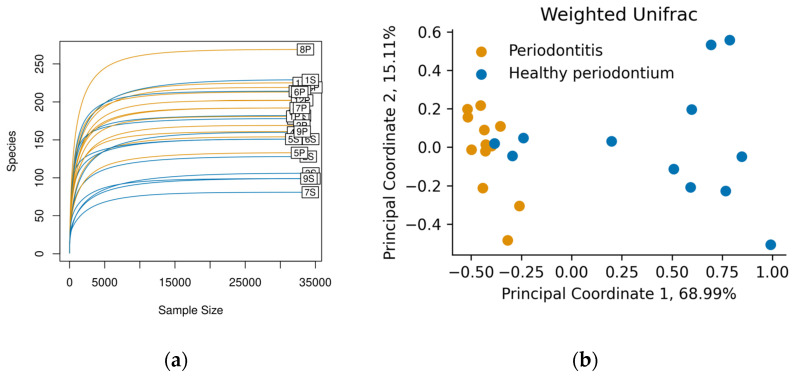
(**a**) Rarefaction curves for each sample show saturation of the identified Amplicon sequence variant (ASVs) (“Species” axis). Figure was made with the vegan R package, version 2.5-7; (**b**) Principal Coordinate Analysis of the Weighted Unifrac diversity metric. Colors represent animals in the Healthy periodontium group (S, Blue) and animals diagnosed with Periodontitis (P, orange).

Swab samples were taken from the gingival margin in the oral cavity. After 16S rRNA sequencing, each sample contained approximately 35,000 reads, and we identified between 80 and 262 ASVs per sample (Figure 1a). The rarefaction curves showed saturation, indicating that the depth of sequencing was appropriate to describe the microbial composition in these groups.

Weighed Unifrac was used to compare the oral microbiome composition in both groups at the phylum level (Figure 1b). There was a clear clustering in the oral microbiome of dogs with periodontal disease, different from dogs with healthy periodontium. This last group showed broader microbiome distributions than diseased animals, who had a more similar microbiome composition (Figure 1b). The difference between groups was corroborated by ANOSIM and PERMANOVA tests (*p* < 0.05).

**Figure 2:**
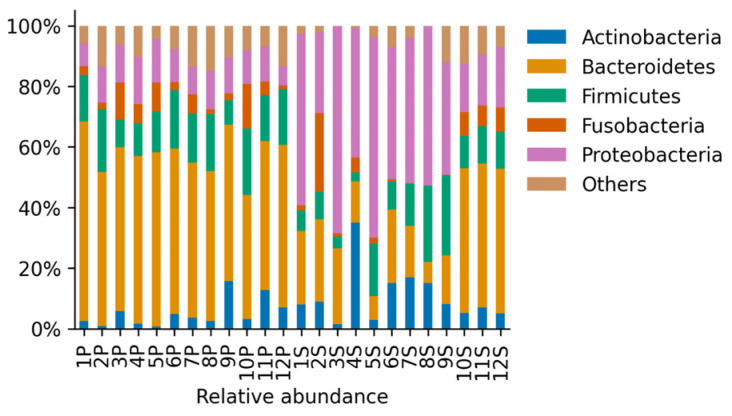
Relative abundance of the identified phyla for samples derived from all animals in the Healthy periodontium group (S, *n* = 12) and in the Periodontitis group (P, *n* = 12). Phyla that were present less than 5% in any sample were aggregated and contributed up to approximately 15% of the relative abundance.

**Figure 3:**
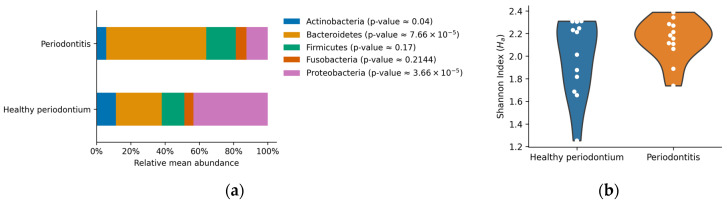
(**a**) Relative mean abundance per group. Bars represent the arithmetic mean for the most abundant phyla (over 5% of the relative abundance). *p*-values of the non-parametric Mann-Whitney *U*-test are shown between parentheses; (**b**) Violin plots of the Shannon diversity index. Each dot represents the determined index per sample, and the shape represents an estimated probability density function of the data.

The most abundant phyla in both groups of animals were *Actinobacteria, Bacteroidetes, Firmicutes*, *Fusobacteria*, and *Proteobacteria* (Figure 2). Other phyla were present below the 5% of the total abundance, contributing 0.08–14.84% in total (Figure 2). Dogs with periodontal disease presented a significant increase in *Bacteroidetes* relative abundance accompanied by a significant reduction in *Actinobacteria* and *Proteobacteria* relative abundance (*p* < 0.05, Figure 3a). These results showed important alterations at the phylum level in the oral microbiome of dogs with periodontal disease. However, no significant differences in the Shannon diversity between healthy and periodontal oral microbiomes were determined (*p* = 0.18; Figure 3b).

**Figure 4:**
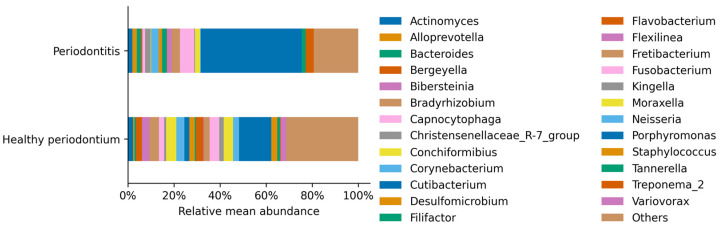
Relative mean abundance of each identified genus per treatment. Genus that was present less than 1% in any sample were aggregated, contributing 31.35% in the healthy periodontium individuals and 19.30% in the group of animals with periodontitis.

The most abundant genus in healthy and periodontal disease dogs was *Porphyromonas*, belonging to the *Bacteroidetes* phylum (Figure 4). The *Porphyromonas* relative abundance increased 2.7 times from 12.9% up to 34.7% of total sequences in periodontal disease, concordant with previous observations (Table 2). This dominance was accompanied by a significant increase in other genera such as *Bacteroides* and *Fusobacterium*, and a significant decrease in less represented genera, such as *Staphylococcus* and *Streptococcus* (Table 2; *p* < 0.05).

**Figure 5:**
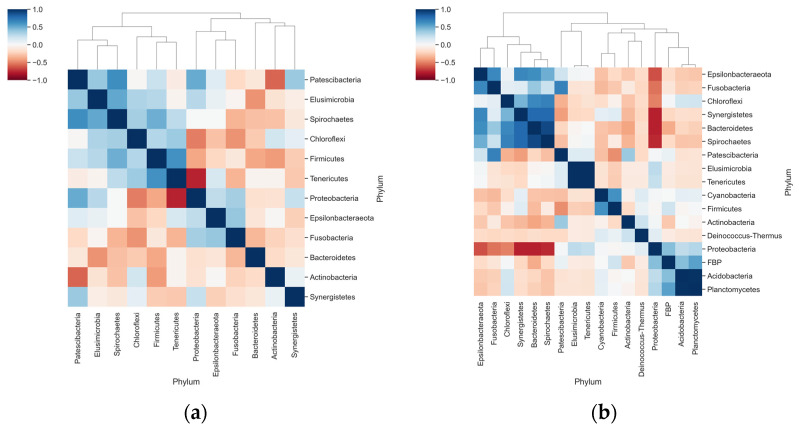
Correlation and clustering analyses for the relative abundance of phyla in each group. (**a**) Heatmap shows the Pearson Correlation Coefficient determined for the relative abundance of phyla only in animals with periodontal disease; (**b**) Pearson correlation coefficients of the relative abundance within animals in the Healthy periodontium group.

Correlation analyses suggested a negative interaction between *Proteobacteria* and *Tenericutes* in dogs with periodontal disease (Figure 5a). Stronger correlations were observed in healthy animals: negative interactions between *Proteobacteria* with *Bacteroidetes* and *Fusobacteria*, and positive correlations in the abundance of Bacteroides with *Synergistetes* and *Spirochetes* (Figure 5b).

**Figure 6:**
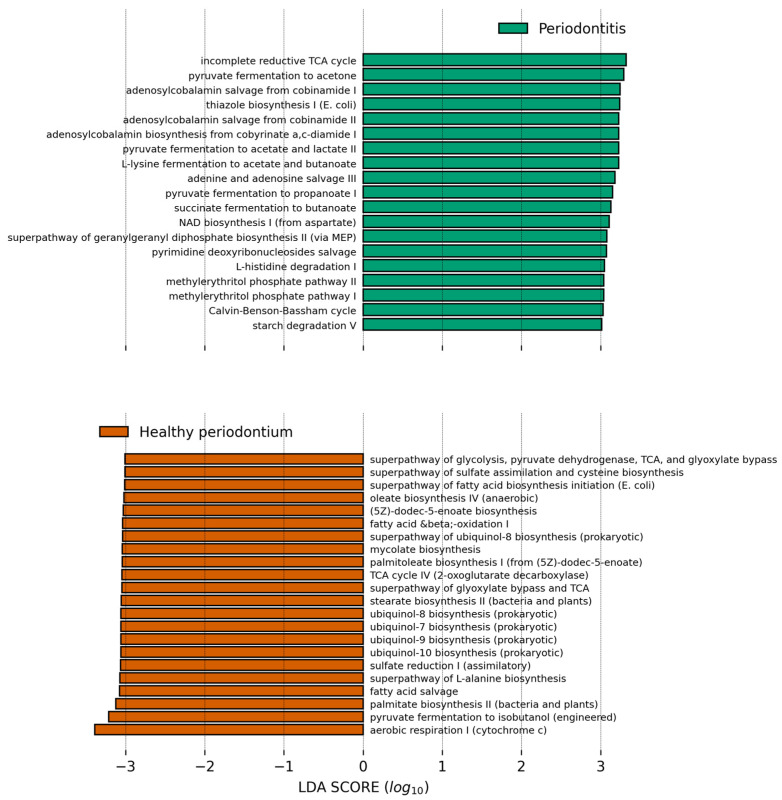
Bar plot showing the PICRUSt2 inferred pathways which relative abundance is higher in the respective group compared to the other and have a Linear Discriminant Analysis score higher than 3.0. Data was analyzed in the Galaxy Server at https://huttenhower.sph.harvard.edu/galaxy/ accessed on 16 October 2021, and results were plotted with a modified script from https://github.com/SegataLab/lefse, accessed on 16 October 2021.

We finally used PICRUSt2 to predict the abundance of major metabolic pathways in the microbiome of both groups and the differential abundance was analyzed with LEfSe (Figure 6). The healthy oral microbiome was enriched in aerobic respiration pathways, especially the glyoxylate bypass and ubiquinol biosynthesis. This enrichment also suggests that aerobic respiration processes are decreased in periodontal disease. The healthy oral microbiome was also enriched in fatty acid biosynthesis (Figure 6). In contrast, functions that are more abundant of the studied animals with periodontal disease compared to animals in the healthy gum group were lipopolysaccharide biosynthesis, coenzyme B12 (adenosylcobalamin) biosynthesis, anaerobic glycolysis, and fermentative processes from pyruvate.

## 4. Discussion

The healthy canine oral microbiome is a diverse, structured community [32]. There is only a small set of studies addressing the composition of the oral microbiome in dogs. This and other studies differ in several aspects, such as the number of animals recruited and sequencing techniques. One essential aspect of studying this community is sampling. It is known that communities in the oral cavity vary significantly across sections in the dental plaque (supragingival, subgingival, saliva) [33,34], however, these studies still have an unrepresentative sample size. Another limitation of this study is the significant difference in the age of the animals, with diseased dogs being older compared to healthy animals. While we cannot rule out that age is a confounding factor in our results, no studies indicate how the oral dog microbiome changes across age, so it would be interesting to have future studies that include animals of different age groups. There is evidence in humans and other models that indicate that the oral microbiome shows a decrease in diversity and increase in taxa, such as *Tannerella* and *Porphyromonas* [35,36]. Important variations in its composition have been observed in dogs compared to healthy oral microbiomes by other studies. It has been suggested that periodontal disease in dogs is associated with a dominance of Gram-positive bacteria [37]. However, this and other studies show that the healthy and periodontal oral microbiome are dominated by two phyla characterized by Gram-negative bacteria, *Proteobacteria* and *Bacteroidetes* [38]. The main outcome observed at the phylum level between groups is an expansion in *Bacteroidetes*, especially *Porphyromonas*, and a reduction in *Proteobacteria.* A modest increase in the *Firmicutes* phylum was also determined in our cohort. A negative correlation between *Proteobacteria* and *Bacteroidetes* sustains the significant changes observed at the phylum level in periodontal disease in dogs.

The canine oral microbiome differs significantly from the human oral microbiome, with only a 16.4% coincidence of bacterial taxa [32]. Likewise, the formation of biofilms in humans has been widely studied, where species in the *Streptococcus* genus have a key role in its development, formation, and maturation of bacterial plaque [39,40,41]. For example, *S. mutans* is characterized by the ability to form biofilms and secrete virulence factors that facilitate caries formation and *S. sanguinis*, known as a pioneer colonizer of oral biofilms [42]. However, it is recognized that in the formation of dental caries there is a dysbiosis of the oral microbiota, where different bacterial species participate that perpetuate and increase this condition [43]. In dogs, the *Streptococcus* genus appears to be subdominant. *Neisseria* and *Corynebacterium* species have also been highlighted in biofilm formation [39]. In this study, we observed that while they have a representation between 2–3% in healthy microbiomes, it is decreased during periodontal disease.

The most abundant genus in both healthy and diseased dogs was *Porphyromonas,* increasing its abundance 2.7 times in diseased dogs. *Porphyromonas* species can modulate the host’s innate immune response processes, resulting in the exacerbation of interleukins, cyclooxygenase 2 directly related to periodontal tissue damage [44,45]. *Porphyromonas gingivalis* is one of the most studied bacteria in humans regarding its development pathways for periodontal disease. It is an opportunistic pathogenic bacterium, which requires anaerobic conditions and nutrients for its growth *in vitro*. It obtains energy by the fermentation of amino acids, which contribute to its growth in inflammatory environments due to the release of products resulting from tissue damage, including peptides. In addition, it can invade gingival epithelial cells, periodontal ligament fibroblasts, immune cells, and osteoblasts [46]. 

This is interesting, since the bacterial lipopolysaccharide (LPS) derived from gram negative bacteria, such as *P. gingivalis*, initiates the acute phase response, thus upregulating the expression of metalloproteinase, increasing the permeability of the gingival epithelium, and altering the humoral immune response, collectively contributing to the periodontal disease [47]. On the other hand, monocytes produce IL-1, TNF-α and β, which induce the destruction of soft tissues of the periodontium, which, associated with the secretion of PGE2 alpha by fibroblasts, generate bone resorption [48].

In dogs, *P. gingivalis* and other bacteria such as *Tannerella forysthia* and *Campylobacter rectus* are part of the periodontal oral microbiome [2,18]. In companion animals such as dogs, bacteria found in the highest proportion in periodontal disease is *Porphyromonas gulae* [38,40,41] and *Porphyromona cangingivalis* [34,49,50]. The striking differences in the oral microbiome of healthy and diseased animals were also reflected in changes in the predicted metabolic functions. Healthy oral microbiomes are characterized by a dominance of aerobic microorganisms (such as Proteobacteria) compared to the microbiomes of dogs with periodontal disease. In the latter, there is an increase in facultative anaerobic and strict anaerobic bacteria such as *Porphyromonas* and *Tannerella* [37]. *Firmicutes* also see an increase in their representation in periodontal disease, which might be favored by a more anaerobic environment, contributing to increments in anaerobic glycolysis and fermentative processes. In diseased dogs, we observed a putative higher production of lipopolysaccharide, a high inflammatory bacterial molecule. Its increase probably contributes to invasion and oral tissue destruction, as it is observed in this disease. In humans, increases in LPS have been related to the origin of the pulp inflammatory process and processes associated with the destruction of alveolar bone in advanced periodontal diseases [51,52].

## Figures and Tables

**Table 1:** Characteristics of the animals (M: male; F: female). Clinical diagnosis was classified in not observed (0) and observed (1). Diet type was Commercial Dry (CD), homemade (HM), and both (CD&HM).

Classification	Code	Sex	Age (y)	Race	Weight (kg)	BS	Diet Type	Gingivitis or Periodontitis	Gingival Index	Dental Calculus	Halitosis	Tooth Exfoliation
Healthy Periodontium (H)	1H	M	2	Mutt	9.8	8	CD&HM	0	0	0	0	0
	2H	F	4	Mutt	29	5	CD&HM	0	0	0	0	0
	3H	M	1	Poodle	7	5	CD	0	0	0	0	0
	4H	M	2	Mutt	10	5	CD&HM	0	0	0	0	0
	5H	F	1	Mutt	20	5	CD&HM	0	0	0	0	0
	6S	F	2.5	Labrador	25	5	CD	0	0	0	0	0
	7H	F	1	German Shepherd	33	5	CD	0	0	0	0	0
	8H	M	7	Teckel	5.8	5	CD	0	0	0	0	0
	9H	M	3	Mutt	17	5	CD	0	0	0	0	0
	10H	M	5	Mutt	21	5	CD&HM	0	0	0	0	0
	11H	F	2	Mutt	18	5	CD&HM	0	0	0	0	0
	12H	M	3	Maltese	5	5	CD	0	0	0	0	0
	Average H		2.79		16.72	5.25						
Periodontitis (P)	1P	F	8	Mutt	35	5	CD	1	1	1	1	0
	2P	M	7	Poodle	6.2	5	CD	1	1	1	1	1
	3P	M	9	Poodle	4.9	5	CD&HM	1	1	1	1	0
	4P	F	3	Mutt	20	5	CD&HM	1	1	1	1	0
	5P	M	4	Mutt	17	5	CD&HM	1	0	1	1	0
	6P	F	3	Cocker Spaniel	12.5	5	CD&HM	1	1	1	1	0
	7P	F	6	Teckel	7	7	CD	1	1	1	1	0
	8P	F	8	Mutt	6	5	CD&HM	1	1	1	1	1
	9P	F	13	Poodle	8.8	7	CD	1	1	1	1	0
	10P	M	8	Maltese	5	5	CD&HM	1	1	1	1	0
	11P	M	6	Chihuahua	4.6	7	CD&HM	1	1	1	1	0
	12P	F	2	Yorkshire	3.5	5	CD&HM	1	1	1	1	0
	Average P		6.42		10.88	5.50		1.00	0.92	1.00	1.00	

**Table 2:** Relative mean abundance of the identified genus in animals on the healthy periodontium (S) and with periodontal disease (*P*) groups. The table shows only the genera with significant changes (*p*-values < 0.05) and ratios different from 0.

Genus	Healthy Periodontium (Mean%)	Periodontitis (Mean%)	Ratio S/P	Ratio P/S	*p*-Value
***Acholeplasma***	0.055 ± 0.19	0.34 ± 0.21	0.16	6.06	0.00116
***Acinetobacter***	0.27 ± 0.44	0.0015 ± 0.0038	177.32	0.01	0.00015
***Alloprevotella***	0.46 ± 0.58	1.7 ± 3.1	0.27	3.74	0.04040
***Bacteroides***	0.62 ± 1.1	1.6 ± 1.7	0.38	2.62	0.01414
***Bosea***	0.1 ± 0.31	0.0031 ± 0.0062	32.16	0.03	0.01828
***Bradyrhizobium***	3.9 ± 6.6	0.007 ± 0.0099	552.06	0.000795	0.00042
***Brevundimonas***	0.079 ± 0.18	0.015 ± 0.015	5.3	0.19	0.03289
***Campylobacter***	0.24 ± 0.41	0.33 ± 0.2	0.73	1.38	0.04374
***Candidatus Tammella***	0.00049 ± 0.0017	0.13 ± 0.14	0.0038	259.03	0.00080
***Catonella***	0.0076 ± 0.024	0.17 ± 0.17	0.05	21.77	0.00024
***Christensenellaceae*** **R-7 group**	0.76 ± 1.1	2 ± 2.3	0.38	2.62	0.02258
***Cutibacterium***	2 ± 3.3	0.0021 ± 0.0051	950.79	0.0011	0.00039
***Defluviitaleaceae*** **UCG-011**	0.16 ± 0.21	0.72 ± 0.46	0.23	4.4	0.00134
***Desulfobulbus***	0.00079 ± 0.0027	0.35 ± 0.52	0.0023	447.73	0.00101
***Desulfoplanes***	0.002 ± 0.0052	0.14 ± 0.19	0.01	69.55	0.04242
***Desulfovibrio***	0.31 ± 0.57	1.3 ± 1.4	0.24	4.12	0.00426
***Ezakiella***	0.021 ± 0.058	0.35 ± 0.51	0.06	16.39	0.00081
***Fastidiosipila***	0.00051 ± 0.0018	0.11 ± 0.12	0.0046	216.92	0.00080
***Filifactor***	0.57 ± 0.75	1.8 ± 1.4	0.32	3.1	0.01018
***Finegoldia***	0.93 ± 1.6	0.00076 ± 0.0026	1233.58	0.00082	0.00253
***Flexilinea***	0.22 ± 0.48	1.8 ± 1.7	0.12	8.01	0.00072
***Fusibacter***	0.39 ± 0.71	0.89 ± 0.64	0.43	2.31	0.01003
***Gemella***	0.39 ± 1	0.0062 ± 0.015	62.64	0.02	0.03913
**H1**	0.027 ± 0.074	0.16 ± 0.12	0.17	5.95	0.00121
***Helcococcus***	0.0051 ± 0.0055	0.47 ± 0.64	0.01	92.4	0.00165
***Luteibacter***	0.1 ± 0.17	0.022 ± 0.0098	4.45	0.22	0.00610
***Massilia***	1.1 ± 3.5	0.055 ± 0.015	19.7	0.05	0.00244
***Odoribacter***	0.0024 ± 0.0062	0.29 ± 0.54	0.01	118.57	0.00012
***Pelomonas***	1.7 ± 3	0.0015 ± 0.0051	1127.24	0.00088	0.00253
***Peptoanaerobacter***	0.19 ± 0.42	0.37 ± 0.35	0.52	1.92	0.03371
***Peptococcus***	0.019 ± 0.043	0.61 ± 0.56	0.03	32.56	0.00008
***Peptoniphilus***	0.27 ± 0.46	0.092 ± 0.22	2.94	0.34	0.04230
***Peptostreptococcus***	0.014 ± 0.018	0.87 ± 1.1	0.02	61.97	0.00086
***Porphyromonas***	13 ± 15	39 ± 11	0.32	3.1	0.00048
***Prevotella*** **7**	0.0022 ± 0.0076	0.24 ± 0.36	0.01	110.08	0.00038
***Propionivibrio***	0.035 ± 0.072	0.092 ± 0.082	0.38	2.63	0.00997
***Proteiniphilum***	0.0022 ± 0.0076	0.22 ± 0.37	0.01	100.8	0.00314
***Proteocatella***	0.2 ± 0.45	0.45 ± 0.56	0.45	2.22	0.01885
***Pseudarthrobacter***	0.39 ± 1.1	0.0075 ± 0.021	51.86	0.02	0.01563
***Pseudomonas***	0.94 ± 2.8	0.073 ± 0.018	12.85	0.08	0.00244
***Rikenellaceae*** **RC9 gut group**	0.0025 ± 0.0059	0.2 ± 0.25	0.01	79.88	0.00015
***Roseburia***	0.0084 ± 0.026	0.22 ± 0.23	0.04	26.23	0.00004
***Ruminiclostridium*** **9**	0.0011 ± 0.0037	0.47 ± 0.93	0.0023	442.97	0.00101
***Ruminococcaceae*** **UCG-004**	0.08 ± 0.21	0.35 ± 0.47	0.23	4.41	0.04291
***Salinisphaera***	0.99 ± 1.5	0.0015 ± 0.0036	647.56	0.0015	0.00280
***Sediminispirochaeta***	0.0049 ± 0.017	0.045 ± 0.045	0.11	9.16	0.00587
***Sphaerochaeta***	0.0081 ± 0.028	0.034 ± 0.071	0.24	4.21	0.04809
***Sphingomonas***	0.13 ± 0.13	0.074 ± 0.017	1.8	0.56	0.01202
***Staphylococcus***	2.4 ± 3.3	0.0027 ± 0.0067	873.18	0.0011	0.00009
***Streptococcus***	1.7 ± 1.9	0.079 ± 0.24	21.19	0.05	0.00203
***Suttonella***	0.16 ± 0.15	0.078 ± 0.028	2.02	0.49	0.04639
***Treponema*** **2**	0.35 ± 0.57	3 ± 1.8	0.12	8.48	0.00019
***Variovorax***	2.2 ± 3.1	0.026 ± 0.024	84.26	0.01	0.00006
***Verticia***	0.12 ± 0.026	0.072 ± 0.028	1.73	0.58	0.00016

## Data Availability

Publicly available datasets were analyzed in this study. This data can be found here https://www.ebi.ac.uk/ena/browser/view/PRJEB47716, accessed on 24 September 2021.
